# Circular RNA ZBTB46 depletion alleviates the progression of Atherosclerosis by regulating the ubiquitination and degradation of hnRNPA2B1 via the AKT/mTOR pathway

**DOI:** 10.1186/s12979-023-00386-0

**Published:** 2023-11-21

**Authors:** Yahong Fu, Qiaowei Jia, Mengmeng Ren, Hengjie Bie, Xin Zhang, Qian Zhang, Shu He, Chengcheng Li, Hanxiao Zhou, Yanjun Wang, Xiongkang Gan, Zhengxian Tao, Xiumei Chen, Enzhi Jia

**Affiliations:** 1 Department of Cardiovascular Medicine, The First Affiliated Hospital of Nanjing Medical University, 210029 Nanjing, Jiangsu Province China; 2 Department of Geriatric, The First Affiliated Hospital of Nanjing Medical University, 210029 Nanjing, Jiangsu Province China; 3 Department of Cardiovascular Medicine, Liyang People’s Hospital, 213300 Changzhou, Jiangsu Province China

**Keywords:** Coronary artery Disease, Atherosclerotic plaque; circular RNA, RNA-binding protein

## Abstract

**Background:**

CircZBTB46 has been identified as being associated with the risk of coronary artery disease (CAD) and has the potential to be a diagnostic biomarker for CAD. However, the specific function and detailed mechanism of circZBTB46 in CAD are still unknown.

**Methods:**

The expression levels and properties of circRNAs were examined using qRT‒PCR, RNA FISH, and subcellular localization analysis. ApoE^−/−^ mice fed a high-fat diet were used to establish an atherosclerosis model. HE, Masson, and Oil Red O staining were used to analyze the morphological features of the plaque. CCK-8, Transwell, and wound healing assays, and flow cytometric analysis were used to evaluate cell proliferation, migration, and apoptosis. RNA pull-down, silver staining, mass spectrometry analysis, and RNA-binding protein immunoprecipitation (RIP) were performed to identify the interacting proteins of circZBTB46.

**Results:**

CircZBTB46 is highly conserved and is significantly upregulated in atherosclerotic lesions. Functional studies revealed that knockdown of circZBTB46 significantly decreased the atherosclerotic plaque area, attenuating the progression of atherosclerosis. In addition, silencing circZBTB46 inhibited cell proliferation and migration and induced apoptosis. Mechanistically, circZBTB46 physically interacted with hnRNPA2B1 and suppressed its degradation, thereby regulating cell functions and the formation of aortic atherosclerotic plaques. Additionally, circZBTB46 was identified as a functional mediator of PTEN-dependent regulation of the AKT/mTOR signaling pathway and thus affected cell proliferation and migration and induced apoptosis.

**Conclusion:**

Our study provides the first direct evidence that circZBTB46 functions as an important regulatory molecule for CAD progression by interacting with hnRNPA2B1 and regulating the PTEN/AKT/mTOR pathway.

**Supplementary Information:**

The online version contains supplementary material available at 10.1186/s12979-023-00386-0.

## Introduction

Coronary artery disease (CAD) is the most widespread type of cardiovascular disease and is predominantly characterized by atherosclerosis of vessel walls. The formation of atherosclerotic plaques may gradually lead to chronic narrowing of arteries and cause ischemic complaints [1]. Clinically, luminal stenosis, arterial blood supply restriction, tissue and organ ischemia and necrosis contribute to clinical events such as myocardial infraction and stroke [2]. As a disease related to aging, CAD is one of the most important cardiovascular diseases affecting human health worldwide and is one of the leading causes of death in both developed and developing countries [3]. Despite considerable progress in treatment approaches for CAD, including coronary artery bypass grafting, percutaneous coronary intervention, and optimal medical therapy, the prevalence of CAD remains stubbornly high, resulting in a heavy socioeconomic burden on individuals and their families [4]. Therefore, it is imperative to elucidate the biological characteristics and molecular mechanisms underlying the development and progression of CAD and develop more efficient diagnostic and therapeutic strategies for CAD.

The cellular of the vasculature is primarily composed of vascular smooth muscle cells (VSMCs) and endothelial cells (ECs), which are components of the tunica media and tunica intima, respectively. These two types of cells can work in a coordinated manner to modulate vascular reactions in direct and indirect ways. In addition, vascular inflammation, endothelial dysfunction, and abnormal vascular smooth muscle cell proliferation/migration are important drivers of the development of atherosclerotic lesions [5–7]. It is known that the pathogenesis of atherosclerosis is very complicated and involves multiple factors, including inflammation, epigenetic modifications, environmental factors, and oxidative stress. Despite several decades of research and technological innovation, the detailed mechanisms of the VSMCs and ECs that compose atherosclerotic lesions have not been clearly delineated.

Circular RNAs (circRNAs), a new class of noncoding RNA without a 5’ cap or 3′ poly(A) tail, are characterized by a closed circular structure, RNA exonuclease resistance, and evolutionary conservation [8]. Emerging studies have shown that circRNAs have profound roles in the occurrence and development of various human diseases, including CAD [9, 10]. However, the molecular mechanisms underlying their regulation in the progression of CAD and atherosclerotic plaques are still largely unexplored. CircZBTB46, located on chromosome 20, is derived from the host gene zinc finger and BTB domain containing 46 (ZBTB46) and is cyclized through a covalent bond formed by the back-splicing of exon 2 and exon 3. A recent study demonstrated that silencing circZBTB46 could inhibit the proliferation and induce the ferroptosis of acute myeloid leukemia (AML) cells and suppress the growth of AML tumors in vivo [11]. Notably, our previous study found that circZBTB46 expression was significantly increased in peripheral blood mononuclear cells (PBMCs) samples from patients with CAD. Furthermore, we revealed that a high circZBTB46 expression level was associated with the incidence of CAD and had positive synergistic effects with hypertension [12]. These findings provide evidence that circZBTB46 may be an important molecular marker and potentially a useful therapeutic target for CAD. Therefore, there is an urgent need to better understand the specific function and detailed mechanism of circZBTB46 in CAD and explore potential therapeutic strategies for CAD.

In the present study, we sought to investigate the influence of circZBTB46 on the progression of atherosclerosis and tried to uncover its underlying mechanism. Our results showed that circZBTB46 exhibited higher expression in human coronary vessels with advanced (stage 4) atherosclerosis than those with early (stage 1) atherosclerosis. Moreover, circZBTB46 expression was significantly increased in a murine atherosclerosis model. Animal studies indicated that silencing circZBTB46 promoted the stabilization of atherosclerotic plaques and inhibited plaque progression. Functional experiments revealed that silencing circZBTB46 inhibited cell proliferation and migration and induced apoptosis in vitro. Mechanistically, circZBTB46 interacted with Heterogeneous Nuclear Ribonucleoprotein A2/B1 (hnRNPA2B1) and stabilized it through a ubiquitin-mediated degradation pathway. Collectively, our results demonstrated that circZBTB46 may be utilized as a potential diagnostic marker or therapeutic target for CAD.

## Materials and methods

### Human coronary artery tissues and cell culture

The human coronary artery tissues used in the present study were obtained from four donors at the Department of Human Anatomy at Nanjing Medical University. The bereaved families of the donors provided written informed consent, and the experiments were reviewed and approved by the ethics committees of Nanjing Medical University and the First Affiliated Hospital of Nanjing Medical University. Details of specimen processing were described in our previous studies [13]. Human coronary artery smooth muscle cells (HCASMCs; Sigma‒Aldrich, USA) and human coronary artery endothelial cells (HCAECs; ScienCell, USA) were cultured with their respective growth media in a humidified incubator with 5% CO_2_ at 37 °C. Cycloheximide (CHX), chloroquine (CQ) and MG132 were purchased from MedChemExpress (New Jersey, USA) and were used for protein stability assays.

### Atherosclerosis mouse model and adeno-associated virus (AAV) intervention

ApoE^−/−^ mice fed a high-fat diet (HFD) have been widely used for the establishment of atherosclerosis (AS) models in vivo [14, 15]. In the present study, seven- to eight-week-old male ApoE^−/−^ mice (GemPharmatech Co., Ltd., Nanjing, China) were fed a HFD (Beijing Keao Xieli Feed Co.,Ltd., Beijing China) to establish an AS model. To investigate the impact of circZBTB46 silencing on the progression of atherosclerotic plaques, mice were injected with AAV9 vectors carrying sh-NC and sh-circZBTB46 (GeneChem Co., Ltd., Shanghai, China) via the tail vein at a titer of 8.0 × 10^11^ in a volume of 200 µl per mouse. After the injection, the mice were continually fed a HFD for another 6 weeks before being sacrificed for tissue collection. All mice were group-housed under SPF conditions at an indoor temperature of 25 ± 2 °C with free access to water and food. The study protocol was approved by the Ethics Committee of the First Affiliated Hospital of Nanjing Medical University. The design of the present study was performed in accordance with the ethical standards laid down in the 1964 Declaration of Helsinki and its later amendments.

### Blood lipid analysis and atherosclerotic lesion analysis

Mice were euthanized by exsanguination at designated time points under anesthesia with isoflurane, and blood samples were collected for blood lipid analysis using triglyceride (TG), total cholesterol (TC), low-density lipoprotein cholesterol (LDL-C) and high-density lipoprotein cholesterol (HDL-C) test kits (Nanjing Jiancheng Bioengineering Institute, Nanjing, China). The hearts and intact aortas from the aortic root to the bifurcation of the iliac artery were then promptly separated and fixed in 4% paraformaldehyde for subsequent pathological analysis. After tissue embedding and sectioning, the sections were stained with hematoxylin and eosin to analyze the morphological features of plaques, Masson staining was used to visualize the collagen fibers, and Oil Red O staining was performed to determine the lipid content. The staining intensity was analyzed by Image-Pro Plus 6.0 software (Media Cybernetics, Inc. Rockville, MD, USA).

### Plasmid and oligonucleotide transfection

The circZBTB46 small interfering RNA (siRNA) oligonucleotides and circZBTB46 overexpression plasmid, along with the corresponding negative controls, were acquired from GenePharma (Shanghai, China), and the hnRNPA2B1 overexpression plasmid was constructed by GeneChem Co., Ltd. (Shanghai, China). The sequences of the siRNAs used in the study are summarized in Supplementary Table 1. Plasmids and siRNAs were transfected into cells using Lipofectamine 3000 Reagent (Invitrogen, Carlsbad, USA) in accordance with the manufacturer’s protocol.

### Fluorescence in situ hybridization (FISH) and subcellular fractionation

FISH was carried out according to the manufacturer’s protocol. The human coronary artery specimen slices were baked at 62 °C for 2 h. The major steps of tissue FISH were as follows: slice dewaxing, rehydration, digestion, prehybridization, hybridization, post-hybridization washes, counterstaining with DAPI, and mounting. Cell culture-treated coverslips were prepared for the in vitro RNA FISH assay. After being rinsed in PBS, the cells were fixed, permeabilized, blocked, and incubated in hybridization buffer with FISH probes. The circZBTB46 and 18 S probes were designed and synthesized by Servicebio (Wuhan, China).

Cytoplasmic and nuclear RNAs molecules were purified using the Cytoplasmic and Nuclear RNA Purification Kit (Norgen Biotek, Ontario, Canada) according to the manufacturer’s instructions. Briefly, cells were lysed by using Lysis Buffer J for 10 min on ice. Then, the lysate was centrifuged for 10 min at maximum speed in a benchtop centrifuge to separate the cell fractions. Subsequently, the cytoplasmic and nuclear RNA were purified and eluted using the respective spin column in accordance with the instructions. The expression level of circZBTB46 was measured by qRT‒PCR, with GAPDH as an internal reference for cytoplasmic RNAs and U6 for nuclear RNAs.

### RNA isolation and quantitative real-time PCR (qRT‒PCR)

First, total RNA was extracted by using Trizol reagent (Vazyme, Nanjing, China), and the concentration and quantification of each sample were measured by a Nanodrop 2000 spectrophotometer (Thermo Fisher Scientific, Waltham, MA, USA). Then, HiScript® III RT SuperMix for qPCR (+ gDNA wiper) (Vazyme, Nanjing, China) was used to synthesize cDNA, which was subsequently amplified by using ChamQ SYBR qPCR Master Mix (Vazyme, Nanjing, China) on a QuantStudio 7 Flex system (Thermo Fisher Scientific, Waltham, MA, USA) for 40 cycles. All primers used are listed in Supplementary Table 2.

### Cell proliferation assay and cell migration assay

Cell proliferation was examined by a Cell Counting Kit 8 assay (CCK-8 kit; APExBIO, USA), while cell migration was assessed by Transwell and wound healing assays. A detailed description of the experimental procedures was presented in our previous articles [16].

### Flow cytometric analysis of apoptosis

According to the instructions of the Annexin V-FITC/PI Apoptosis Detection Kit (Vazyme, Nanjing, China), cells were detached with EDTA-free trypsin, and then, 1 × 10^5^ cells were harvested after centrifugation at 1000 rpm, 4 °C for 5 min. Afterward, the cells were washed twice with precooled PBS at 1000 rpm and 4 °C for 5 min and then resuspended in 100 µl of 1× Binding Buffer. Subsequently, the cells were incubated with 5 µl of Annexin V-FITC and 5 µl of propidium iodide (PI) staining solution in the dark at room temperature (20–25 °C) for 10 min. Finally, 400 µl of 1× Binding Buffer was added to each tube, and the stained samples were detected by flow cytometry (FACScan, BD Biosciences, CA, USA) within 1 h.

### Western blot analysis

RIPA lysis buffer containing 1 mM PMSF reagent (Beyotime, Shanghai, China) was used to extract total protein from cell samples. Protein concentrations in the lysates were then quantified by a bicinchoninic acid assay (Beyotime, Shanghai, China) after sonication and centrifugation. Then, loading buffer was added to the lysates, and proteins were denatured at 95° C for 10 min. Subsequently, proteins were separated on SDS‒PAGE gels and transferred onto a polyvinylidene difluoride (PVDF) membrane (0.22 μm, Millipore, USA), which was then blocked with 5% skim milk and subjected to immunoblot analysis. Finally, an ECL Chemiluminescence Kit (Vazyme, Nanjing, China) was used to visualize the protein bands through signal detection. The anti-hnRNPA2B1, anti-CyclinD1, anti-β-actin, anti-α-tubulin, anti-Ubiquitin, anti-AKT, anti-mTOR, and anti-p-mTOR antibodies were purchased from Proteintech (Wuhan, China); the anti-cleaved caspase 3 antibody was obtained from Cell Signaling Technology (Danvers, MA, USA); the anti-cleaved PARP and anti-GAPDH antibodies were obtained from HUABIO (Hangzhou, China); and the anti-p-AKT, anti-Cyclin A and anti-PTEN antibodies were obtained from Wanlei Biotechnology Company (Shenyang, China).

### RNA pull-down, silver staining, and mass spectrometry analysis

The biotin-labeled circZBTB46 probe and antisense were synthesized by GenePharma (Shanghai, China), and RNA pull-down assay was performed using a Pierce Magnetic RNA-Protein Pull-Down Kit (Thermo Fisher Scientific, Waltham, MA, USA). In brief, RNA probe (100 pmol) was mixed with 50 ul prewashed streptavidin magnetic beads and incubated for 30 min at room temperature with agitation. Then, the magnetic beads were washed with an equal volume of 20 mM Tris (pH 7.5), and 100 µL of 1X Protein-RNA Binding Buffer was added to the beads and mixed well. Subsequently, RNA-Protein Binding Reaction Master Mix was prepared and added to the RNA-bound beads, and the mixture was then incubated for 60 min at 4 °C with agitation. Finally, RNA-binding protein complexes were eluted with 50 µL of Elution Buffer and separated on SDS‒PAGE gels, and the gels were then subjected to silver staining (Beyotime, Shanghai, China). The selected bands were identified by mass spectrometry (BiotechPack, Beijing, China) and Western blot analysis.

### RNA-binding protein immunoprecipitation (RIP) assays

Under the guidance of the manufacturer’s instructions, we performed RIP assays by using an EZ-Magna RIP Kit (Millipore, Billerica, USA). Briefly, 5 µg of the anti-hnRNPA2B1 antibody and negative control IgG (Millipore, Billerica, USA) were incubated with magnetic beads for 30 min at room temperature with agitation. Then, the supernatant of the cell lysate was incubated overnight at 4 ℃ with antibody-conjugated magnetic beads for each RIP reaction. Finally, proteins in each sample were digested by proteinase K, and RNA was purified for subsequent downstream analysis.

### Modeling of the circZBTB46‑hnRNPA2B1 interaction

The secondary structure of circZBTB46 was predicted via the online web server RNAfold [17] (http://rna.tbi.univie.ac.at/cgi-bin/RNAWebSuite/RNAfold.cgi). Then, the 3dRNA/DNA Web Server [18] (http://biophy.hust.edu.cn/new/3dRNA) and I-TASSER [19] (https://zhanggroup.org/I-TASSER/) were used to predict the three-dimensional structures of circZBTB46 and hnRNPA2B1, respectively. Moreover, the ubiquitination sites in the hnRNPA2B1 protein were predicted via a Bayesian discriminant method (http://bdmpub.biocuckoo.org/prediction.php). In addition, the interaction sites in circZBTB46 and hnRNPA2B1 were identified with catRAPID [20] (http://service.tartaglialab.com/page/catrapid_group) and the HDOCK server [21] (http://hdock.phys.hust.edu.cn/) and was then visualized using Discovery Studio 2021 (client version).

### Immunohistochemical staining (IHC)

We conducted immunohistochemical staining in accordance with a standard protocol, which included deparaffinization, rehydration, antigen retrieval, and blocking. Then, the sections were incubated with a primary antibody at 4 °C overnight and subsequently incubated with a secondary antibody for 30 min at room temperature prior to color development with DAB. After counterstaining with hematoxylin, the slides were dehydrated, transparentized using xylene, and mounted with neutral resin.

### Coimmunoprecipitation (Co-IP) assay

We performed a co-IP assay using an Immunoprecipitation Kit with Protein A + G Magnetic Beads (Beyotime, Shanghai, China) according to the manufacturer’s instructions. Protein A/G beads were first incubated with the anti-hnRNPA2B1 antibody at room temperature for 2 h with agitation. Then, cell lysates were added to the protein A/G bead–antibody complexes and incubated overnight at 4 °C with rotation. Finally, SDS‒PAGE Sample Loading Buffer (1X) was used to elute precipitated proteins at 95 °C for 5 min, and the supernatants were collected for WB analysis with anti-hnRNPA2B1 or anti-ubiquitin (Proteintech, Wuhan, China) antibodies.

### Statistical analysis

Statistical analysis of the data in the present study was performed by GraphPad Prism 8, and the data are presented as the means ± SEMs. Comparisons between two groups were conducted by two-tailed Student’s t test for normally distributed data or the Kruskal-Wallis test for nonnormally distributed data, while comparisons among more than two groups were conducted by one-way ANOVA test. A *p* value < 0.05 was considered to indicate statistical significance.

## Results

### CircZBTB46 is highly expressed in CAD

**Fig. 1:**
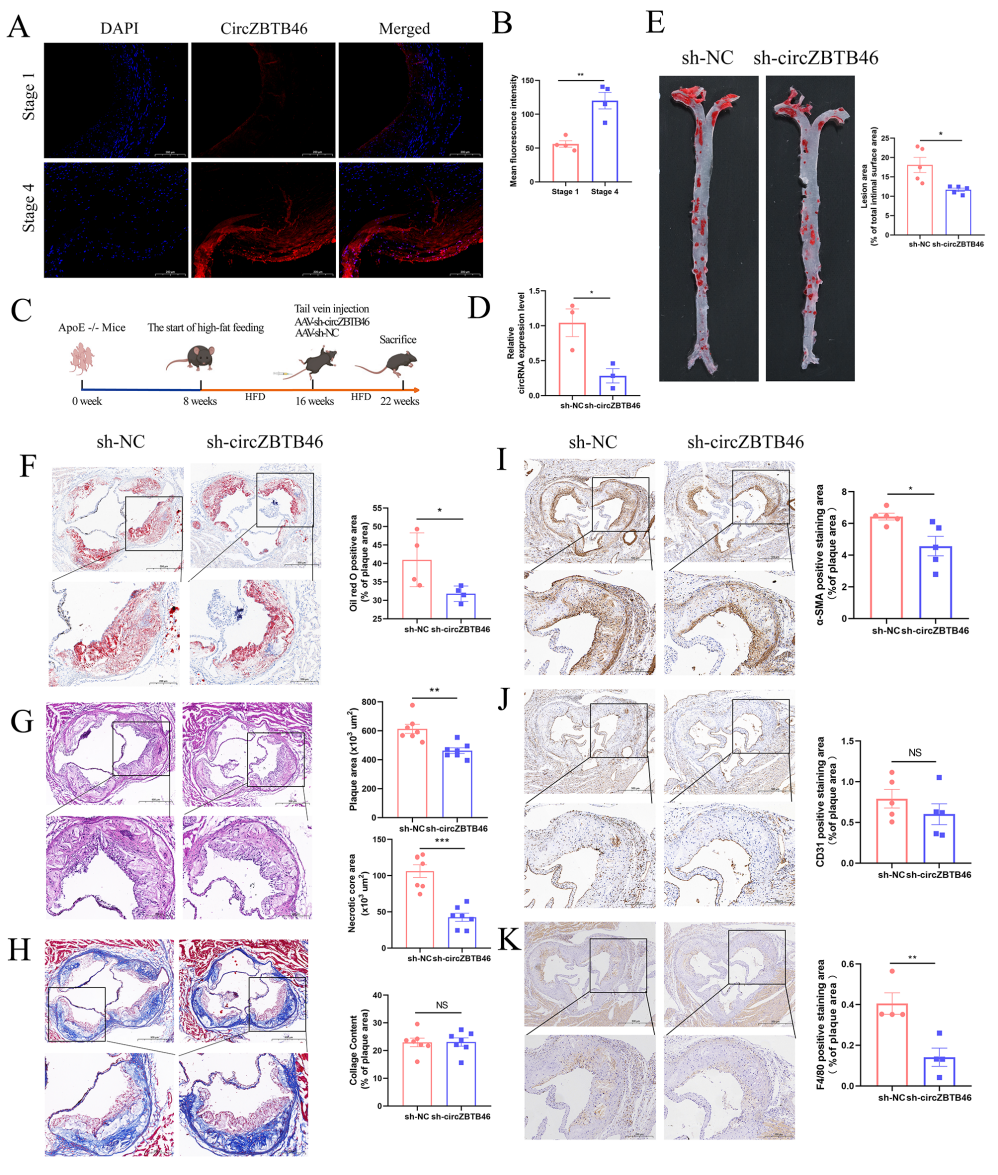
CircZBTB46 is highly expressed in CAD, and circZBTB46 knockdown attenuates the progression of atherosclerosis. **(A-B)** Fluorescence in situ hybridization (FISH) was used to show the location and expression of circZBTB46 in human atherosclerotic plaques. Fluorescence intensity was measured using ImageJ software and the mean fluorescence intensity was represented as mean ± SEM. **(C) **The schedule of atherosclerosis model establishment and AAV intervention. **(D)** The relative expression of circZBTB46 in the heart in the two groups. **(E-F)** Oil red O staining of the intact aortas and cross-sections of aortic roots. **(G)** HE staining of the aortic sinus in the AAV-sh-NC and AAV-sh-circZBTB46. **(H)** Masson staining of the aortic sinus in the two groups of mice after AAV intervention. **(I)** α-SMA, (J) CD31, and **(K)** F4/80 staining in the aortic sinus in the AAV-sh-NC and AAV-sh-circZBTB46 groups. *p < 0.05, **p < 0.01, ***p < 0.001

In our previous study, circZBTB46 was found to be upregulated in PBMC samples of CAD patients and was identified as a potential biomarker for CAD [12]. Here, we verified that circZBTB46 exhibited higher expression in human coronary vessels with advanced (stage 4) atherosclerosis than those with early (stage 1) atherosclerosis by using FISH **(**Fig. 1A-B**)**. Furthermore, we found that circZBTB46 was abundantly expressed in smooth muscle cells and endothelial cells. (Supplementary Fig. 1A-B). In addition, circZBTB46 was identified to be evolutionarily conserved, with a sequence identity of 84.22% (1057/1255) between humans and mice (Supplementary Fig. 1C). In addition, we established an atherosclerosis model by using ApoE^−/−^ mice fed a high-fat diet. The results of Oil red O and HE staining showed that compared with those in the C57BL/6 control mice, the plaque areas were significantly increased in the atherosclerosis model mice (Supplementary Fig. 1D-E). We then investigated the expression of the murine circZBTB46 homolog (mmu_circ_0009684) in heart tissues of the murine model of atherosclerosis. CircZBTB46 was expressed at a higher level in the atherosclerosis model group than in the control group (Supplementary Fig. 1F).

### Silencing circZBTB46 attenuates the progression of Atherosclerosis in vivo

To investigate the impact of circZBTB46 silencing on the progression of atherosclerotic plaques, mice were injected with AAV9 vectors carrying sh-NC and sh-circZBTB46 via the tail vein. The schedule of atherosclerosis model establishment and AAV intervention is shown in Fig. 1C. After 6 weeks of AAV intervention, the expression of circZBTB46 was significantly decreased in the AAV-sh-circZBTB46 groups compared with the AAV-sh-NC groups **(**Fig. 1D**)**. Oil red O staining of intact aortas and cross-sections of aortic roots showed that circZBTB46 inhibition reduced the size of atherosclerotic plaque lesions **(**Fig. 1E-F**)**. In addition, HE staining of the aortic sinus suggested that the plaque size and necrotic core area were significantly reduced in AAV-sh-circZBTB46 mice. However, the Masson staining results indicated that there was no significant difference in the collagen content between the two groups **(**Fig. 1G-H**)**. Immunohistochemical staining was performed to quantify staining for α-SMA (the marker for smooth muscle cells), CD31 (the marker for endothelial cells) and F4/80 (the marker gene for macrophages) in the aortic plaques. The results revealed that the areas of positive α-SMA and F4/80 staining were significantly decreased in AAV-sh-circZBTB46 mice **(**Fig. 1I-K**)**. In addition, there were no significant effects of AAV-sh-circZBTB46 treatment on the average body weight or the levels of HDL-C, LDL-C, TC and TG (Supplementary Fig. 1G-H).

### **Silencing circZBTB46 inhibits cell proliferation and migration and induces apoptosis in vitro**

**Fig. 2:**
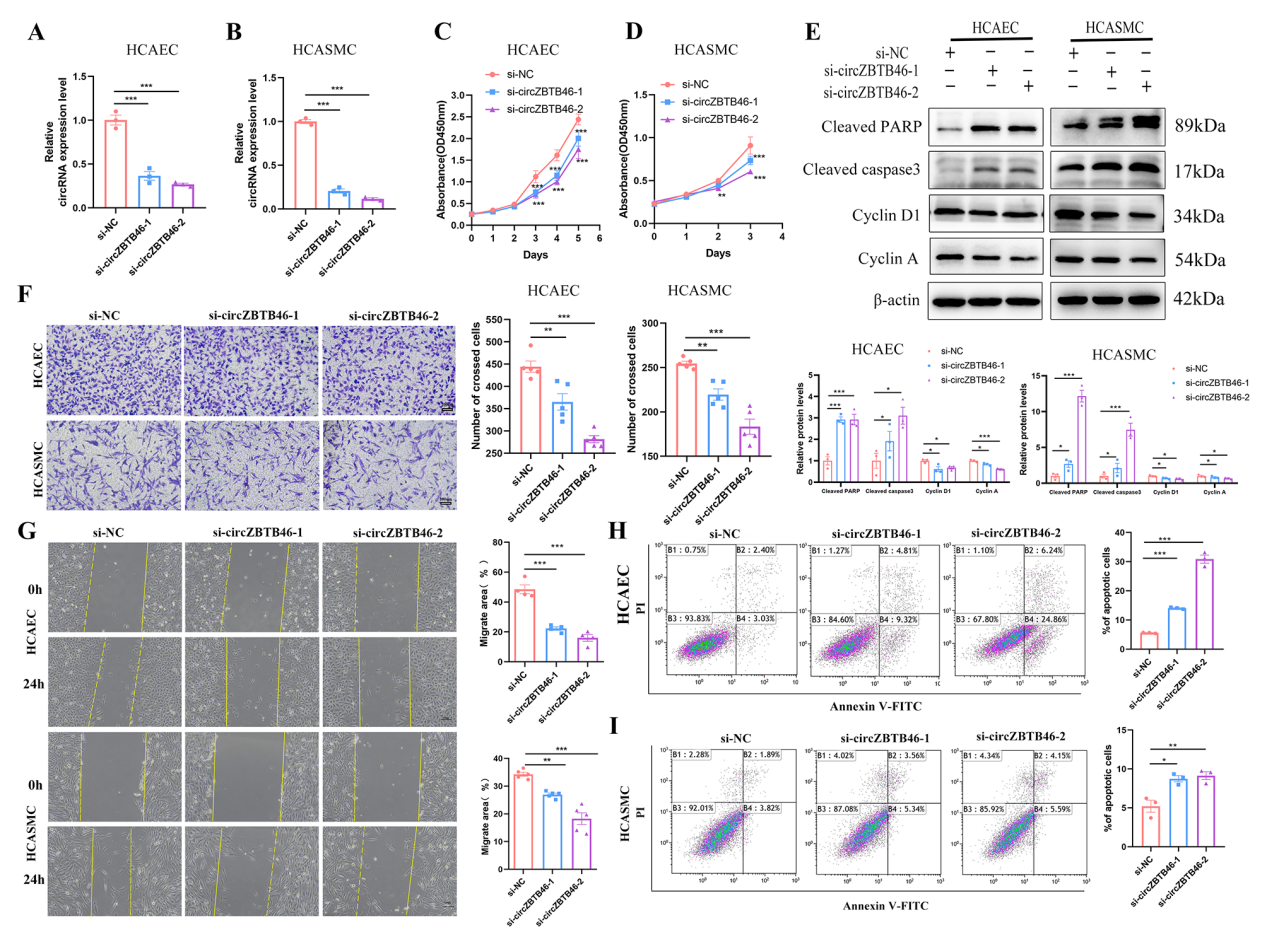
Silencing circZBTB46 inhibits cell proliferation and migration and induces apoptosis. **(A-B)** The relative expression of circZBTB46 in HCAECs and HCASMCs transfected with si-NC, si-circZBTB46-1, and si-circZBTB46-2. **(C-D)** The CCK-8 assay revealed that circZBTB46 knockdown inhibited cell proliferation. **(E)** Western blot analysis showed that the protein levels of cleaved PARP and cleaved caspase 3 were significantly increased whereas, Cyclin D1 and Cyclin A were significantly decreased upon silencing of circZBTB46. **(F-G)** The migration of HCAECs and HCASMCs was inhibited by knockdown of circZBTB46. **(H-I)** Flow cytometric analysis was performed to determine showed the percentages of apoptotic cells after transfection with si-NC, si-circZBTB46-1, and si-circZBTB46-2. *p < 0.05, **p < 0.01, ***p < 0.001

Given the crucial roles of endothelial cells and smooth muscle cells in the development and progression of CAD, we examined the expression of circZBTB46 in HCAECs and HCASMCs and investigated the role of circZBTB46 through loss-of-function studies. We designed and synthesized two siRNAs targeting the back-splicing junction of circZBTB46, and both significantly reduced the expression of circZBTB46 in vitro **(**Fig. 2A-B**)**. Cell Counting Kit-8 (CCK-8) assays demonstrated that silencing circZBTB46 significantly decreased the proliferative capacity of HCAECs and HCASMCs **(**Fig. 2C-D**)**. Furthermore, as demonstrated by transwell and wound healing assays, circZBTB46 knockdown also decreased cell migration **(**Fig. 2F-G**)**. Additionally, the percentage of apoptotic cells was dramatically increased upon the transfection of si-circZBTB46, determined through flow cytometric analysis **(**Fig. 2H-I**)**. Moreover, Western blot analysis showed that the protein levels of cleaved PARP and cleaved caspase 3 were significantly increased whereas those of Cyclin D1 and Cyclin A were significantly decreased upon silencing of circZBTB46 **(**Fig. 2E**)**.

### CircZBTB46 physically interacts with hnRNPA2B1

**Fig. 3:**
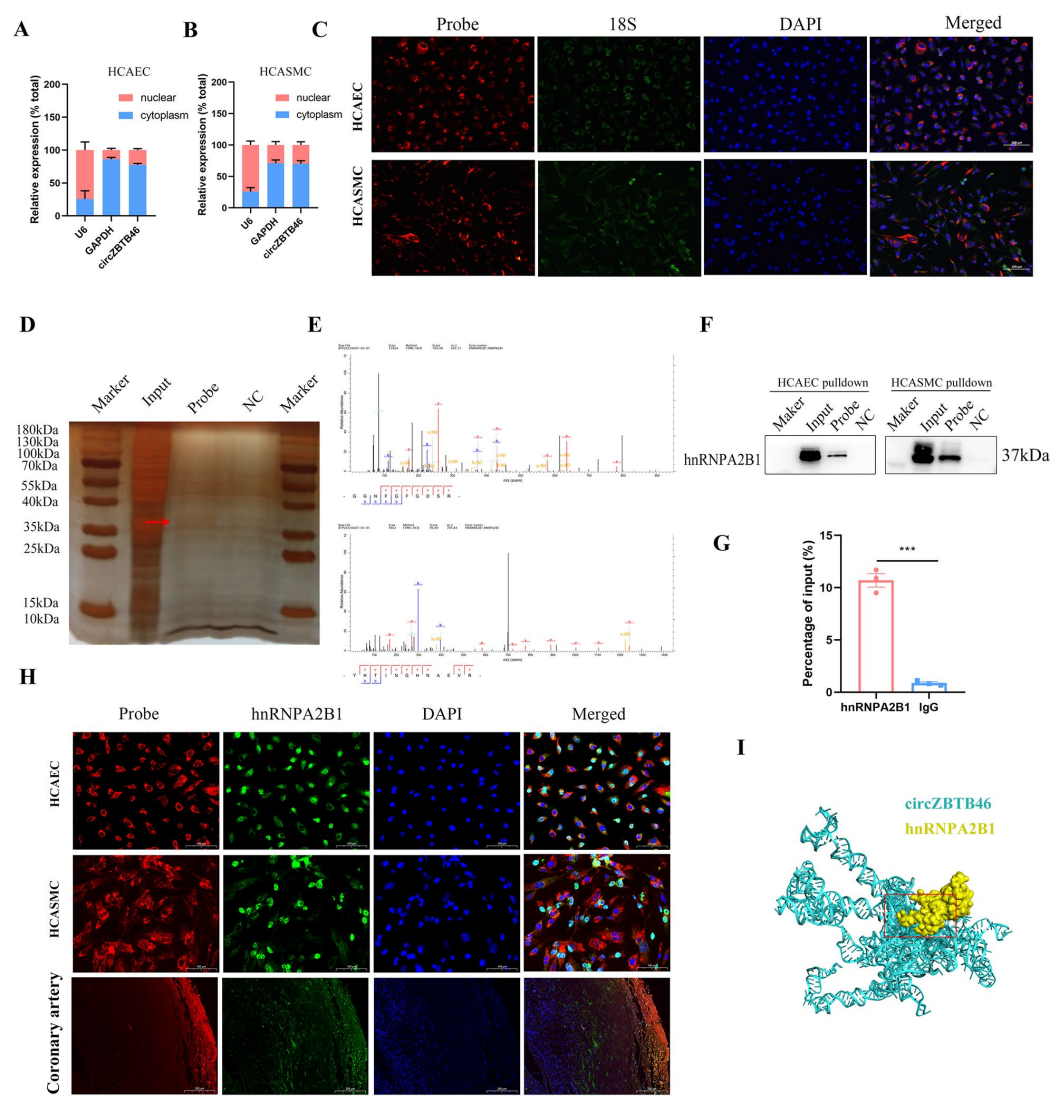
CircZBTB46 physically interacts with hnRNPA2B1. **(A-C)** The relative subcellular localization of circZBTB46 was assessed by qRT‒PCR and FISH. **(D)** Silver staining of the gel after SDS‒PAGE separation of the proteins that were immunoprecipitated with the 3′ biotin-labeled probe for circZBTB46 (probe) and the biotinylated antisense probe (NC) in RNA pull-down assays. The arrow shows the position of hnRNPA2B1. **(E)** Mass spectrometry analysis showed that hnRNPA2B1 was the top-ranked protein according to the peptide identification results. **(F) **Western blot analysis following the RNA pull-down assay and **(G)** qRT‒PCR analysis following the RIP assay confirmed the interaction between circZBTB46 and hnRNPA2B1. **(H)** FISH showed that circZBTB46 was colocalized with hnRNPA2B1 in cells and human coronary arteries. **(I)** 3‑Dimensional structure of the circZBTB46‑hnRNPA2B1 complex

We next investigated the specific molecular mechanisms by which circZBTB46 functions in CAD. First, the subcellular localization of circZBTB46 was determined by RNA nuclear-cytoplasmic fractionation and fluorescence in situ hybridization (FISH). We found that circZBTB46 was distributed in both the nucleus and the cytoplasm, but the cytoplasm exhibited much more robust accumulation **(**Fig. 3A-C**)**. In addition, 3′ biotin-labeled probes for circZBTB46 (probe) and a biotinylated antisense probe (NC) were designed for RNA pull down assay to identify the potential protein interaction partners of circZBTB46. After silver staining, we found a specific band at approximately 37 kDa in the probe group compared with the NC group. Subsequently, mass spectrometry analysis showed that 33 proteins were significantly upregulated in the probe group, with hnRNPA2B1 ranked the highest according to the peptide identification results **(**Fig. 3D-E**).** Synchronously, proteins pulled down by the circZBTB46 and antisense probes were subjected to immunoblot analysis with an anti-hnRNPA2B1 antibody. Notably, the results again confirmed the direct binding of hnRNPA2B1 and circZBTB46 **(**Fig. 3F**)**. Importantly, RNA immunoprecipitation assays further confirmed that circZBTB46 was markedly enriched in the hnRNPA2B1 precipitate compared with the IgG precipitate **(**Fig. 3G**)**. Furthermore, we evaluated the colocalization of circZBTB46 and hnRNPA2B1 by FISH in human coronary arteries and two kinds of cells. The results indicated the colocalization of circZBTB46 with hnRNPA2B1, which corroborated that circZBTB46 indeed bound to hnRNPA2B1 **(**Fig. 3H**).** Together, these data indicate that hnRNPA2B1 might specifically interact with circZBTB46 and may also act as a protein scaffold to enable circZBTB46 to mediate the expression of its downstream target genes. Additionally, we further predicted the binding sites in hnRNPA2B1 and circZBTB46 with the catRAPID and HDOCK servers, and then visualized them using Discovery Studio 2021 (client version) **(**Fig. 3I**)**. Bioinformatics analysis with catRAPID revealed that the hnRNPA2B1-circZBTB46 interaction was primarily mediated through the nucleotides 901–952, 905–956, 505–556, 105–156, and 101–152 in the RNA and through protein domains of 166–217, 66–117, 116–167, 51–102, and 101-152aa.

#### Silencing hnRNPA2B1 inhibits cell proliferation and induces apoptosis in vitro

**Fig. 4:**
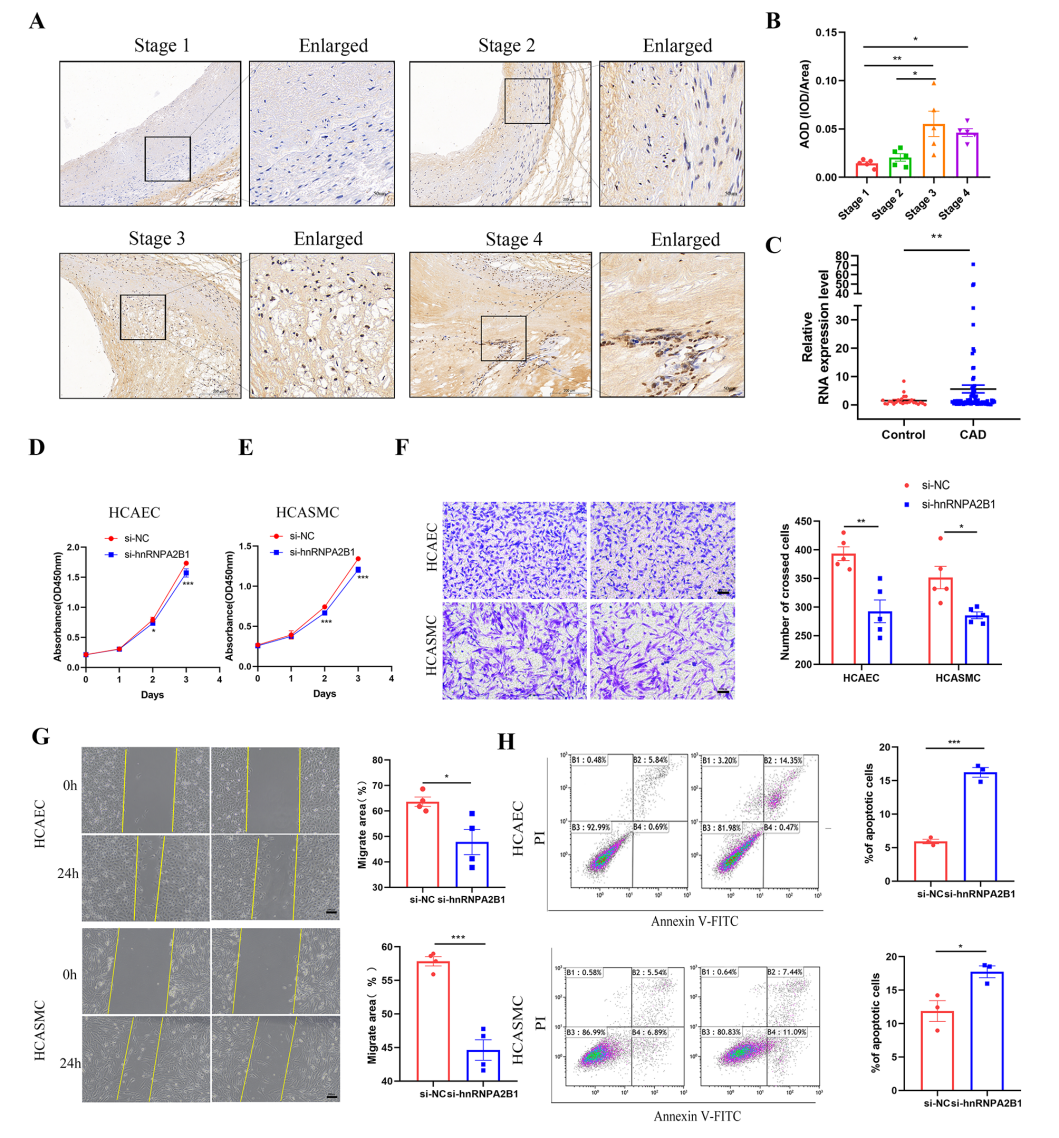
hnRNPA2B1 is upregulated in atherosclerotic plaques, and silencing hnRNPA2B1 inhibits cell proliferation and induces apoptosis. **(A-B)** The expression of hnRNPA2B1 in different stages of atherosclerosis was analyzed by immunohistochemical staining. **(C)** The relative expression levels of hnRNPA2B1 in PBMC samples from CAD patients and controls were determined by RT‒PCR. **(D-E)** The CCK-8 assay revealed that silencing hnRNPA2B1 inhibited cell proliferation. **(F-G)** Transwell and wound healing assays suggested that cell migration was dramatically suppressed by silencing hnRNPA2B1 expression. **(H)** Flow cytometric analysis indicated that the proportion of apoptotic cells was notably elevated in the si-hnRNPA2B1 groups. *p < 0.05, **p < 0.01, ***p < 0.001

Previous studies confirmed that hnRNPA2B1 plays crucial roles in the proliferation, migration, and drug resistance of multiple types of tumor cells [22–24]. However, whether the hnRNPA2B1 level is associated with CAD—in other words, whether hnRNPA2B1 can affect the functions of HCAECs and HCASMCs—remains unclear. First, we analyzed the expression of hnRNPA2B1 in human coronary artery segments with different stages of atherosclerosis by immunohistochemical staining. The results demonstrated a correlation between hnRNPA2B1 expression and atherosclerotic plaques. As shown in Fig. 4A-B, the expression level of hnRNPA2B1 was significantly higher in stage 3 and stage 4 atherosclerotic plaques. We then investigated the expression of hnRNPA2B1 in PBMC samples from CAD patients and controls by qRT‒PCR. Consistently, we found a trend toward higher expression of hnRNPA2B1 mRNA in patients with CAD than in controls **(**Fig. 4C**).** Additionally, we designed specific siRNAs targeting hnRNPA2B1 to investigate the role of hnRNPA2B1 in vitro. The transfection efficiency of each siRNA is shown in Supplementary Fig. 2A-B. CCK-8 assays revealed that cell proliferation was significantly attenuated after transfection with si-hnRNPA2B1 **(**Fig. 4D-E**)**. Furthermore, transwell and wound healing assays demonstrated that cell migration was dramatically suppressed by silencing hnRNPA2B1 expression **(**Fig. 4F-G**)**. Conversely, flow cytometric analysis indicated that the proportion of apoptotic cells was notably elevated in the si-hnRNPA2B1 group **(**Fig. 4H**)**. These results suggested that hnRNPA2B1 not only is a CAD-associated gene but also regulates several biological processes in HCAECs and HCASMCs.

### CircZBTB46 inhibits cell proliferation and migration through hnRNPA2B1 and the PTEN/AKT/mTOR pathway

**Fig. 5:**
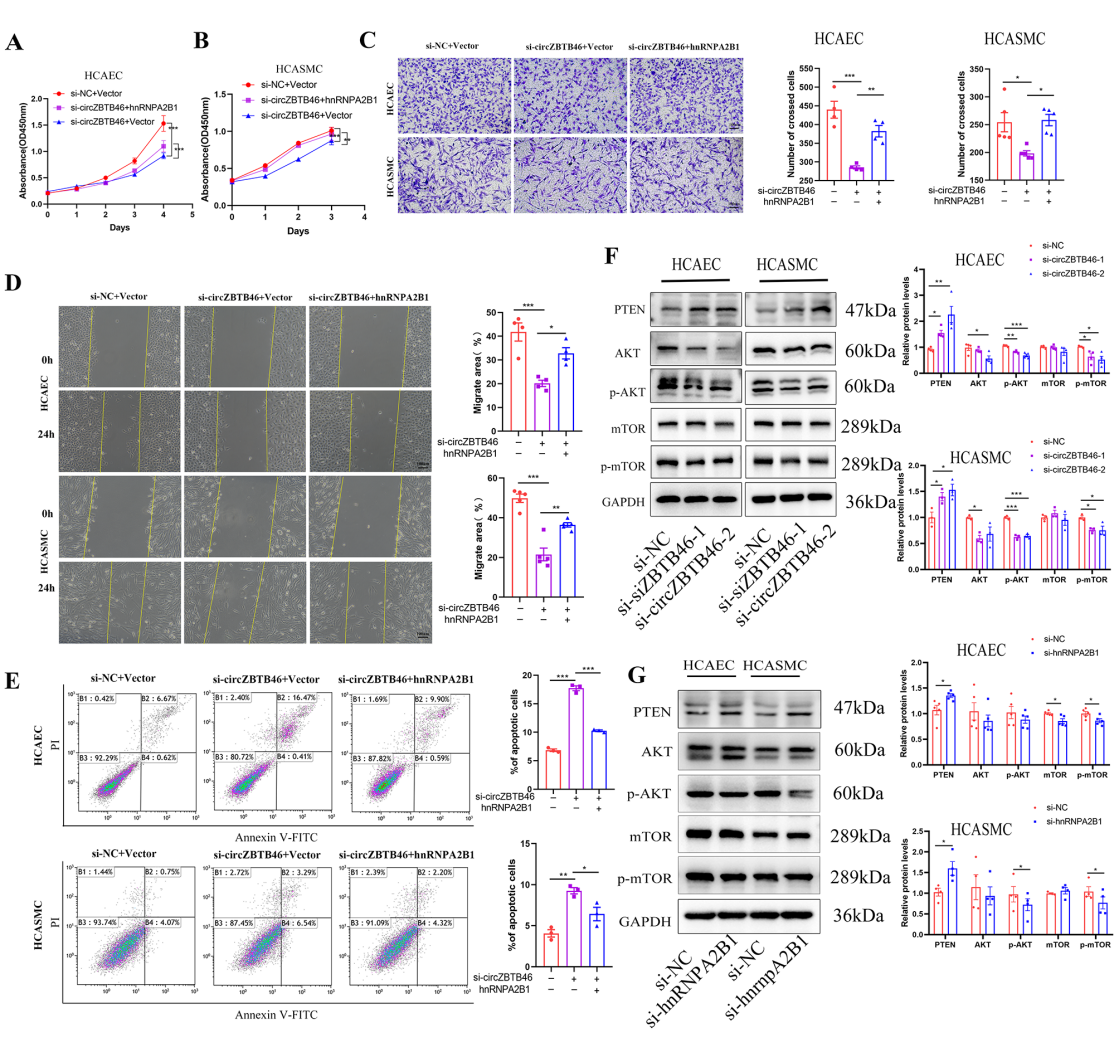
CircZBTB46 inhibits cell proliferation and migration through hnRNPA2B1 and the PTEN/AKT/mTOR pathway. **(A-D)** Overexpression of hnRNPA2B1 attenuated the inhibitory effects of circZBTB46 knockdown on the proliferation and migration of HCAECs and HCASMCs. **(E)** Overexpression of hnRNPA2B1 partially blocked circZBTB46-silencing induced cell apoptosis. **(F-G)** Silencing circZBTB46 or hnRNPA2B1 increased the expression of PTEN and inhibited AKT and mTOR phosphorylation. *p < 0.05, **p < 0.01, ***p < 0.001

To further elucidate the function of hnRNPA2B1 in the circZBTB46-induced biological effects, we transfected siRNAs to knock down endogenous circZBTB46 while simultaneously overexpressing hnRNPA2B1 in HCAECs and HCASMCs. Rescue experiments revealed that overexpression of hnRNPA2B1 attenuated the inhibitory effects of circZBTB46 knockdown on the proliferation and migration of HCAECs and HCASMCs **(**Fig. 5A-D**)**. Moreover, flow cytometric analysis indicated that overexpression of hnRNPA2B1 partially blocked circZBTB46 silencing-induced apoptosis **(**Fig. 5E**)**. Collectively, these results suggested that overexpression of hnRNPA2B1 partially abolished the effects of circZBTB46 knockdown on cell proliferation, migration, and apoptosis.

During the development of diseases, upstream target genes can activate signaling cascades through signaling pathways, changing the cellular phenotype. It has been reported that the PI3K/AKT/mTOR signaling pathway plays important biological roles in the processes of cellular growth, survival, proliferation, apoptosis, angiogenesis, and metabolism [25]. This pathway has been implicated as a key pathway in regulating the formation of atherosclerotic plaques [26, 27]. To explore the specific mechanism by which circZBTB46 modulates these biological processes, we further conducted functional enrichment analyses of the proteins pulled down by circZBTB46. Gene Ontology analysis revealed that the circZBTB46-interacting proteins are involved in multiple biological processes. Pathway analysis suggested that the mTOR signaling pathway was enriched in the circZBTB46-interacting proteins (Supplementary Fig. 3A-B). Particularly, emerging evidence has demonstrated that hnRNPA2B1 regulates tumor proliferation and metastasis through the regulation of the AKT/mTOR signaling pathway [23, 28, 29]. We therefore investigated the roles of circZBTB46 and hnRNPA2B1 in the AKT/mTOR pathway. The results indicated that circZBTB46 knockdown increased the expression of PTEN and inhibited AKT and mTOR phosphorylation. Since hnRNPA2B1 is a functional downstream target of circZBTB46, we monitored its effects on the regulation of the AKT/mTOR pathway. Western blot analysis demonstrated that silencing hnRNPA2B1 also led to the inhibition of the PTEN/AKT/mTOR signaling pathway. **(**Fig. 5F-G**)**. Thus, we hypothesized that circZBTB46 could act as a functional mediator of PTEN-dependent regulation of the AKT/mTOR signaling pathway and thus affect cell proliferation and migration and induce apoptosis.

#### CircZBTB46 blocks the ubiquitination and degradation of hnRNPA2B1

**Fig. 6:**
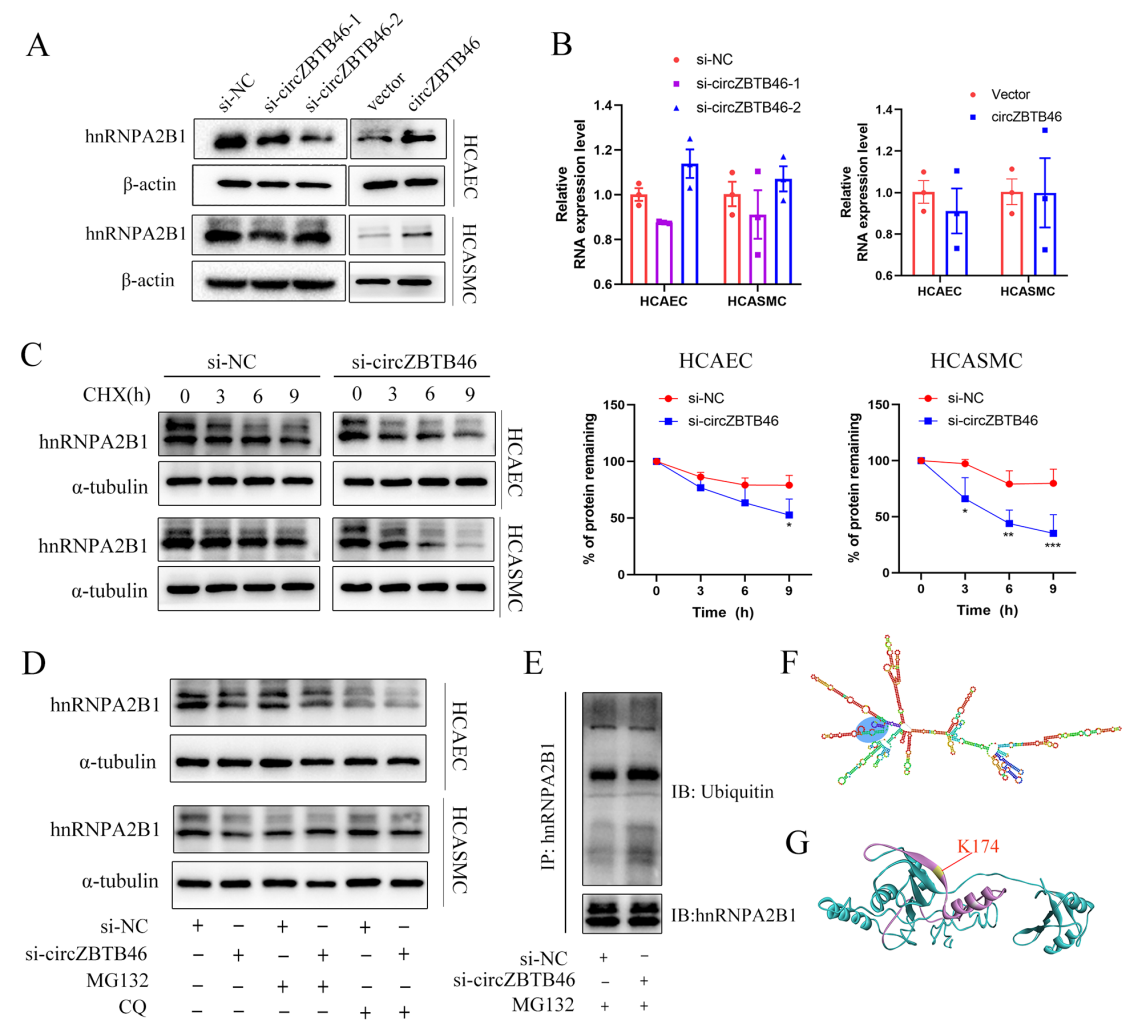
CircZBTB46 blocks the ubiquitination and degradation of hnRNPA2B1. **(A)** Western blot analysis of the expression level of hnRNPA2B1 after circZBTB46 knockdown or circZBTB46 overexpression. **(B)** RT‒PCR analysis of hnRNPA2B1 expression after transfection with si-circZBTB46 or the circZBTB46 overexpression plasmid. **(C) **Immunoblot analysis of the hnRNPA2B1 level in intact cells treated with CHX for 0, 3, 6 or 9 h. The relative fold changes were determined by comparison with the level at 0 h. **(D)** Cells transfected with si-circZBTB46 were treated with MG132 or CQ, and the hnRNPA2B1 level in the indicated cells was measured by Western blotting. **(E)** Lysates from circZBTB46-knockdown cells were immunoprecipitated (IP) with an anti-hnRNPA2B1 antibody and were then used for immunoblot analysis of ubiquitin and hnRNPA2B1. **(F)** Secondary structure prediction of circZBTB46 using the online web server RNAfold. The predicted binding sites of circZBTB46 with hnRNPA2B1 are shown in the blue circle. **(G)** The ubiquitination sites in hnRNPA2B1 were predicted via a Bayesian discriminant method and visualized using Discovery Studio 2021 (client version). Purple indicates the binding region, while yellow indicates the ubiquitination site

Based on the literature reports, mechanistic insights have been gained rough knowledge of the roles of other ncRNAs that act as protein interaction partners [30–33]. We reasoned that circZBTB46 might interact with hnRNPA2B1 and regulate its expression at the transcriptional, posttranscriptional, or posttranslational levels to regulate biological functions. Therefore, we further investigated both the effect of circZBTB46 on hnRNPA2B1 expression and the specific molecular mechanisms. Specifically, we overexpressed or knocked down circZBTB46 in vitro. We then investigated the relationship between circZBTB46 and hnRNPA2B1 expression. The WB results that suggested silencing of circZBTB46 in vitro caused significant downregulation of hnRNPA2B1 protein expression, whereas overexpression of circZBTB46 increased the hnRNPA2B1 protein level (Fig. 6A). However, qRT‒PCR analysis suggested that circZBTB46 modulation had no effect on the mRNA level of hnRNPA2B1, indicating that the effect of circZBTB46 on hnRNPA2B1 expression is controlled by a posttranscriptional mechanism **(**Fig. 6B).

Additionally, we further treated HCAECs and HCASMCs with cycloheximide (CHX, 20 µg/ml) after transfection with si-circZBTB46 to determine the regulatory effects of circZBTB46 on the stability of hnRNPA2B1. Notably, we found that circZBTB46 knockdown significantly shortened the half-life of hnRNPA2B1 (Fig. 6C). Moreover, the presence of the proteasome inhibitor MG132 but not the lysosomal inhibitor chloroquine (CQ) effectively abrogated the downregulation of hnRNPA2B1, indicating that circZBTB46 might regulate hnRNPA2B1 via a proteasome-dependent pathway. Besides, the co-IP assay results suggested that knockdown of circZBTB46 increased hnRNPA2B1 ubiquitination. **(**Fig. 6D-E**)**. Moreover, we predicted the possible ubiquitination sites in hnRNPA2B1 in the PDM-PUB database. Through combined analysis of the interaction site in circZBTB46-hnRNPA2B1 predicted by catRAPID, we found that the 901–952 nucleotides region in circZBTB46 was predicted to bind to 166-217aa domains of hnRNPA2B1 protein and block the attachment of the K174-linked ubiquitin chain **(**Fig. 6F-G**)**. In addition, when we predicted the proteins that may bind to hnRNPA2B1 via the HitPredict database [34–36] (http://www.hitpredict.org), we found that 15 E-3 ubiquitin protein ligases were predicted to bind with high confidence (Supplementary Fig. 4), further suggesting that hnRNPA2B1 may be degraded via ubiquitination. According to these findings, we speculated that circZBTB46 performed its function through blocking the ubiquitination and degradation of hnRNPA2B1.

## Discussion

CircRNAs, as a novel class of non-coding RNAs with considerable biological implications, have critical regulatory roles in cardiovascular diseases [37]. Currently, biological functions and regulatory mechanisms have been elucidated for only a few circRNAs [38]. In the present study, we investigated the roles and action mechanisms of circZBTB46 in regulating cellular functions and the progression of atherosclerotic plaques. Our results indicated that circZBTB46 is highly conserved and is significantly upregulated in atherosclerotic plaques in both humans and mice. Importantly, knockdown of circZBTB46 significantly reduced the plaque lipid content, decreased the plaque and necrotic core areas, and attenuated the progression of atherosclerosis. Functional experiments suggested that silencing circZBTB46 inhibited cell proliferation and migration and induced apoptosis. Mechanistically, we proposed a model in which circZBTB46 physically interacts with hnRNPA2B1 and inhibits its degradation, thereby regulating cell functions and the formation of aortic atherosclerotic plaques. Additionally, circZBTB46 was identified as a functional mediator of PTEN-dependent regulation of the AKT/mTOR signaling pathway, thus affecting cell proliferation, migration and inducing apoptosis.

CAD is the most common cardiovascular disease and poses a serious threat to human health, and its mortality rate continues to increase annually. Vascular endothelial cells (ECs) and smooth muscle cells (SMCs) play important roles not only in vascular homeostasis but also in the development of diseases [39]. Since atherosclerosis is the major cause of CAD, exploring the biological functions and regulatory mechanisms of CAD risk genes in human atherosclerosis is particularly important for revealing the pathogenesis and identifying molecular biomarkers and therapeutic targets for CAD [40]. Zhang found that circ_0086296 is upregulated in human carotid artery plaques and can promote ECs injury and atherosclerosis progression via an IFIT1/STAT1 feedback loop by sponging miR-576-3p [41]. Moreover, hsa_circ_0030042 was found to be expressed at a low level in human atherosclerotic plaques, and overexpression of exogenous hsa_circ_0030042 improved plaque stability [15]. Hsa_circ_0000280 was also reported to be downregulated in human atherosclerotic vessels. That study further confirmed that hsa_circ_0000280 reduced neointimal thickness and smooth muscle cell proliferation by interacting with ELAVL1 [42]. Our data revealed that circZBTB46 is highly conserved and is significantly upregulated in atherosclerotic plaques in both humans and mice. These results were also in agreement with our previous report that circZBTB46 expression was significantly increased in patients with CAD and may be a potential diagnostic biomarker for CAD [12]. Here, we confirmed that silencing circZBTB46 could inhibit cell proliferation and migration, and induce apoptosis by interacting with hnRNPA2B1 and that circZBTB46 is involved in the progression of atherosclerotic plaques. This result provides new evidence for further exploration of the roles of circRNAs in the progression of CAD and improves the understanding of the pathogenic mechanism of CAD.

Prior studies have reported that circRNAs can act as miRNA sponges to regulate gene expression, as scaffolds for RNA-binding proteins, and even as templates for protein or short peptide translation [8, 38]. The present study demonstrated that circZBTB46 could act as a protein scaffold, providing binding sites for a specific interaction with hnRNPA2B1 and thus mediating the expression of its downstream target genes. The hnRNPA2B1 protein is a member of the heterogeneous nuclear ribonucleoproteins (hnRNPs) family, which is reported to be involved in a variety of biological processes, such as mRNA processing, splicing and transport; DNA repair; and regulation of gene expression [43, 44]. Previous reports regarding the cellular function of hnRNPA2B1 have focused on its role in cancers. Studies have found that hnRNPA2B1 is abnormally expressed in various cancers, indicating that it may be a potential screening biomarker for cancer [43]. For example, hnRNPA2B1 is a potential therapeutic target in multiple myeloma through its mediation of the expression of AKT3 [23]. Knockdown of hnRNPA2B1 restored tamoxifen and fulvestrant sensitivity in breast cancer cells and was involved in AKT and MAPK pathway activation [24]. Moreover, the lncRNA MIR100HG was found to facilitate TCF7L2 mRNA stability and colorectal cancer progression by interacting with hnRNPA2B1 [22].

To date, very few studies have investigated the biological function of hnRNPA2B1 in CAD. To directly assess the clinical relevance of hnRNPA2B1 in CAD and atherosclerotic plaque, we further investigated hnRNPA2B1 protein expression in human coronary artery segments with different stages of atherosclerosis by immunohistochemical staining and measured the hnRNPA2B1 mRNA level in PBMCs of patients with CAD as well as controls. Our results demonstrated that the expression level of hnRNPA2B1 was significantly increased in advanced atherosclerotic plaques. Consistently, we found a trend toward higher expression of hnRNPA2B1 mRNA in patients with CAD than in controls. In addition, functional experiments showed that silencing hnRNPA2B1 inhibited cell proliferation and induced apoptosis in vitro and that exogenous overexpression of hnRNPA2B1 reversed the inhibitory effects of circZBTB46 knockdown on the proliferation and migration of HCAECs and HCASMCs. Mechanistically, we demonstrated that circZBTB46 could stabilize the hnRNPA2B1 protein at the posttranscriptional level by blocking its ubiquitination and degradation. In agreement with a prior report, we identified multiple ubiquitination sites in hnRNPA2B1 and hypothesized that the ubiquitin–proteasome pathway is an important mechanism for its degradation [45]. In support of these findings, bioinformatics analysis revealed that the 901–952 nucleotides region in circZBTB46 was predicted to bind with 166-217aa domains in the hnRNPA2B1 protein and block the K174-linked ubiquitin chains. In addition, we predicted 15 high-confidence E-3 ubiquitin protein ligases that may bind to hnRNPA2B1 via the HitPredict database, a further indication that hnRNPA2B1 may be degraded via ubiquitination. Since our study showed that circZBTB46 expression was inversely correlated with hnRNPA2B1 ubiquitination, we speculate that circZBTB46 may also act as a scaffold for hnRNPA2B1 and regulate the ubiquitination process by competitively binding hnRNPA2B1 with E3 ubiquitin protein ligases. However, additional experiments are still warranted to delineate the precise molecular mechanisms.

During the development of diseases, upstream target genes can activate signaling cascades through signaling pathways, changing the cellular phenotype. The AKT/mTOR pathway is a classical intracellular signaling pathway, and its abnormal activation or dysfunction can affect the pathological processes of various cardiovascular diseases [46–48]. PTEN has been reported to be a key endogenous negative regulatory factor of the AKT/mTOR pathway [49, 50]. Increasing evidence has shown that circRNAs can act as upstream molecules of the AKT/mTOR pathway and participate in the progression of diseases [51, 52]. Moreover, many researchers have demonstrated that hnRNPA2B1 regulates tumor proliferation and metastasis through regulation of the AKT/mTOR signaling pathway [23, 28, 29]. Additionally, pathway analysis suggested that the mTOR signaling pathway was enriched in circZBTB46-interacting proteins. Therefore, we sought to investigate the role of circZBTB46 in regulating the AKT/mTOR signaling pathway. Our results showed that circZBTB46 knockdown increased the expression of PTEN and inhibited AKT and mTOR phosphorylation, thereby inhibiting the activation of the AKT/mTOR signaling axis. Admittedly, we regard our findings as preliminary and await more detailed future analyses. Here, we report only that circZBTB46 and hnRNPA2B1 can affect the expression of PTEN and regulate the levels of AKT and mTOR phosphorylation, and the specific regulatory site and the downstream targets still need further experimental verification. However, these data could provide additional evidence for unveiling novel mechanisms in CAD progression.

## Conclusion

The pathogenesis of atherosclerosis is a progressive, complex process, associated with diverse molecular changes, and atherosclerosis can be serious and lethal when it progresses to a late stage. Despite advances in CAD treatment, the residual risk of CAD remains high. Exploring the biological functions and regulatory mechanisms of CAD risk genes in human atherosclerosis is particularly important for revealing the pathogenesis of and identifying molecular biomarkers and therapeutic targets for CAD. Although substantial progress has been made in research on the pathogenic mechanisms of circRNAs associated with CAD, many challenges remain. Our findings revealed that circZBTB46 is highly conserved and is significantly upregulated in atherosclerotic plaques in both humans and mice. Importantly, knockdown of circZBTB46 significantly reduced the plaque lipid content, decreased the plaque and necrotic core areas, and attenuated the progression of atherosclerosis. The results highlighted circZBTB46 as a promising therapeutic target that plays an important role in atherosclerosis progression by interacting with hnRNPA2B1 and regulating its ubiquitination and degradation. Our findings provide further scientific evidence and new perspectives for future studies on the therapeutic targets of CAD.

### Electronic supplementary material

Below is the link to the electronic supplementary material.

Supplementary Material 1

Supplementary Material 2

Supplementary Material 3. Supplementary Fig. 1. CircZBTB46 is evolutionarily conserved and highly expressed in CAD. (A) The locations of circZBTB46 and α-SMA were determined by FISH. (B) The locations of circZBTB46 and CD31 were determined by FISH. (C) Structural diagram and Sanger sequencing of circZBTB46 across human and mouse species. (D) Oil red O staining of intact aortas and (E) HE staining of aortic sinuses in C57BL/6 and ApoE^−/−^ mice. (F) The relative expression levels of circZBTB46 in C57BL/6 and ApoE^−/−^ mice. (G-H) The average body weight and the levels of HDL-C, LDL-C, TC and TG in mice in the AAV-sh-NC and AAV-sh-circZBTB46 groups.

Supplementary Material 4. Supplementary Fig. 2. RT‒PCR and Western blot analysis for quantifying hnRNPA2B1 expression after transfection with siRNAs./

Supplementary Material 5. Supplementary Fig. 3. GO and KEGG pathway analysis of circZBTB46-interacting proteins.

Supplementary Material 6. Supplementary Fig. 4. Potential E-3 ubiquitin-protein ligases of hnRNPA2B1 predicted by the HitPredict Database.

## Data Availability

All data and materials have been made available.

## Abbreviations

CAD: coronary artery disease
HE: Hematoxylin and eosin
WB: western blot
RIP: RNA-binding protein immunoprecipitation
VSMCs: vascular smooth muscle cells
ECs: endothelial cells
circRNAs: Circular RNAs
ZBTB46: zinc finger and BTB domain containing 46
AMI: acute myeloid leukemia
PBMCs: peripheral blood mononuclear cells
hnRNPA2B1: Heterogeneous Nuclear Ribonucleoprotein A2/B1
HCASMCs: Human coronary artery smooth muscle cells
HCAECs: Human coronary artery endothelial cells
CHX: cycloheximide
CQ: chloroquine
AAV: adeno-associated virus
HFD: high-fat diet
AS: atherosclerosis
TG: Triglyceride
TC: total cholesterol
LDL-C: low-density lipoprotein cholesterol
HDL-C: high-density lipoprotein cholesterol
siRNA: small interfering RNA
FISH: Fluorescence in situ hybridization
qRT‒PCR: quantitative real-time PCR
PVDF: polyvinylidene difluoride
IHC: Immunohistochemical
Co-IP: Co-immunoprecipitation
hnRNPs: heterogeneous nuclear ribonucleoproteins
