# Supplementary material for: Circular RNA ZBTB46 depletion alleviates the progression of Atherosclerosis by regulating the ubiquitination and degradation of hnRNPA2B1 via the AKT/mTOR pathway
Source: Immun Ageing. 2023 Nov 21;20:66. doi: 10.1186/s12979-023-00386-0 (PMC10662463; doi:10.1186/s12979-023-00386-0)
Supplement: Supplementary file 1 — Supplementary Material 1 [file 12979_2023_386_MOESM1_ESM.docx]

Supplementary Table 1. The sequences of siRNAs.

| siRNA name | Sense (5'-3') | Antisense (5'-3') |
| --- | --- | --- |
| Si-circZBTB46-1 | CCACUCGCUGUCCCAGUCUTT | AGACUGGGACAGCGAGUGGTT |
| Si-circZBTB46-2 | UCGCUGUCCCAGUCUGUAGTT | CUACAGACUGGGACAGCGATT |
| Si-hnRNPA2B1-1 | GGCUUUGUCUAGACAAGAATT | UUCUUGUCUAGACAAAGCCTT |
| Si-hnRNPA2B1-2 | GCUGCAAGACCUCAUUCAATT | UUGAAUGAGGUCUUGCAGCTT |
| Si-hnRNPA2B1-3 | CAGGAAGUUCAGAGUUCUATT | UAGAACUCUGAACUUCCUGTT |
