# Supplementary material for: Circular RNA ZBTB46 depletion alleviates the progression of Atherosclerosis by regulating the ubiquitination and degradation of hnRNPA2B1 via the AKT/mTOR pathway
Source: Immun Ageing. 2023 Nov 21;20:66. doi: 10.1186/s12979-023-00386-0 (PMC10662463; doi:10.1186/s12979-023-00386-0)
Supplement: Supplementary file 2 — Supplementary Material 2 [file 12979_2023_386_MOESM2_ESM.docx]

Supplementary Table 2. Primers used in RT-PCR.

| Primer name | Forward primer (5'->3') | Reverse primer (5'->3') |
| --- | --- | --- |
| hsa_circZBTB46 | CGGCGCTCATGAGTAAGAAC | CGCCTCTTCTACAGACTGGG |
| hsa_hnRNPA2B1 | GTGGACGTGGATTTGGGGAT | CATAACCACCTCCGTAGCCC |
| hsa_GAPDH | AAGGTCGGAGTCAACGGATT | ATGGAATTTGCCATGGGTGG |
| hsa_U6 | CTCGCTTCGGCAGCACA | AACGCTTCACGAATTTGCGT |
| mmu-circZBTB46 | TGGCTCACTTCTGTTCGAGT | TCGGTTGTTCATCTGGGAGT |
| mmu_GAPDH | CTGCGACTTCAACAGCAACT | TCATTGTCATACCAGGAAATGAGC |
