# Supplementary figures and images for: Circular RNA ZBTB46 depletion alleviates the progression of Atherosclerosis by regulating the ubiquitination and degradation of hnRNPA2B1 via the AKT/mTOR pathway
Source: Immun Ageing. 2023 Nov 21;20:66. doi: 10.1186/s12979-023-00386-0 (PMC10662463; doi:10.1186/s12979-023-00386-0)

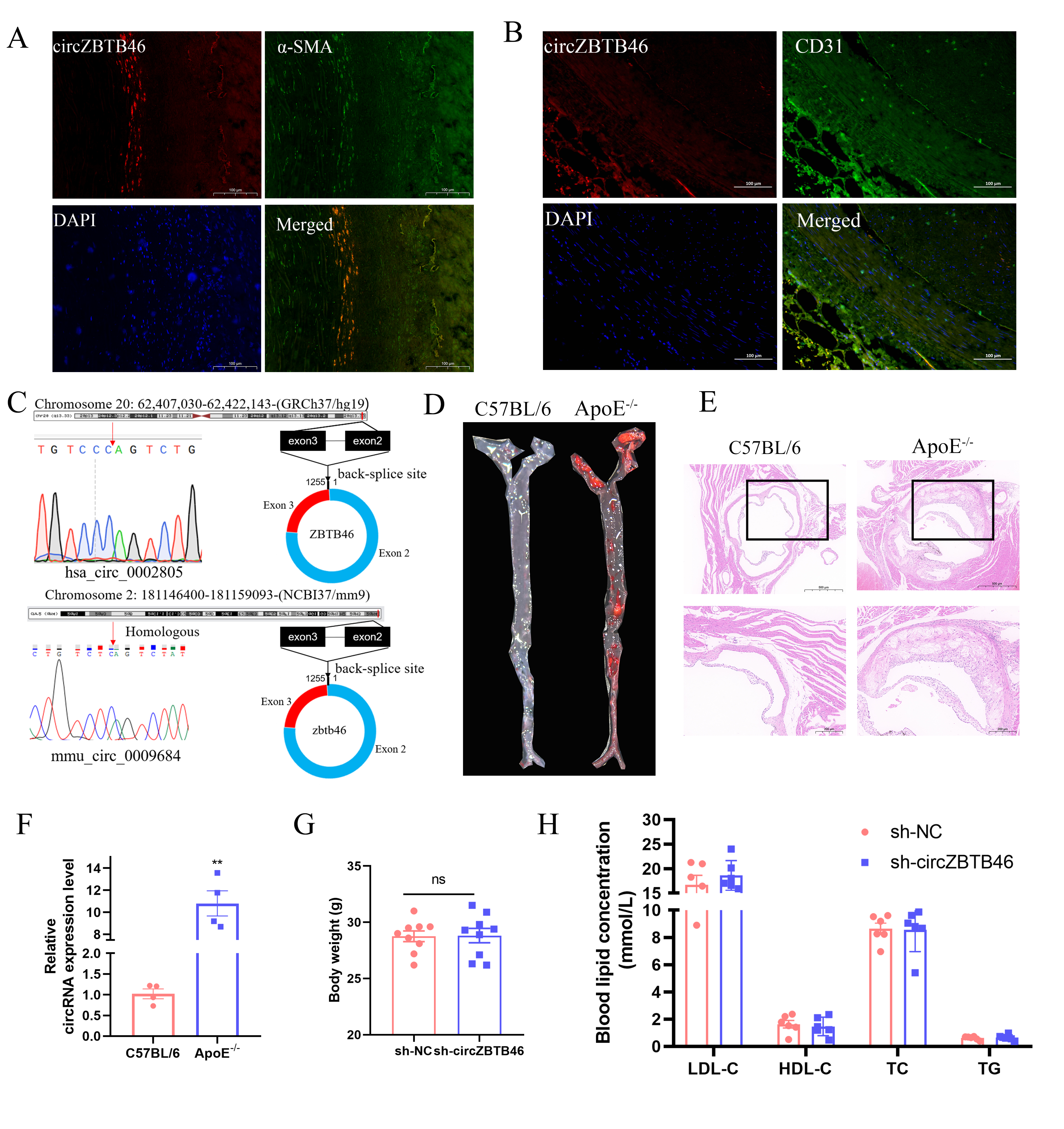

Supplement: Supplementary file 3 — Supplementary Material 3. Supplementary Fig. 1. CircZBTB46 is evolutionarily conserved and highly expressed in CAD. (A) The locations of circZBTB46 and α-SMA were determined by FISH. (B) The locations of circZBTB46 and CD31 were determined by FISH. (C) Structural diagram and Sanger sequencing of circZBTB46 across human and mouse species. (D) Oil red O staining of intact aortas and (E) HE staining of aortic sinuses in C57BL/6 and ApoE−/− mice. (F) The relative expression levels of circZBTB46 in C57BL/6 and ApoE−/− mice. (G-H) The average body weight and the levels of HDL-C, LDL-C, TC and TG in mice in the AAV-sh-NC and AAV-sh-circZBTB46 groups. [file 12979_2023_386_MOESM3_ESM.tif]

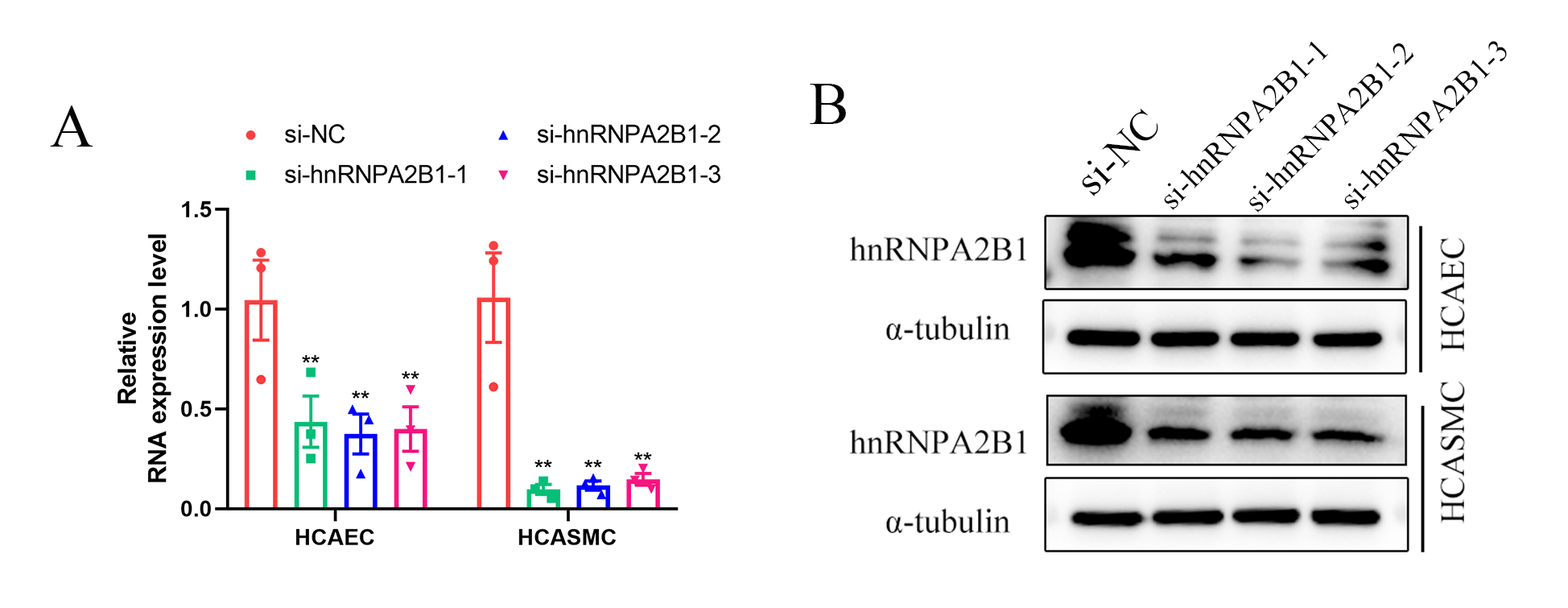

Supplement: Supplementary file 4 — Supplementary Material 4. Supplementary Fig. 2. RT‒PCR and Western blot analysis for quantifying hnRNPA2B1 expression after transfection with siRNAs./ [file 12979_2023_386_MOESM4_ESM.tif]

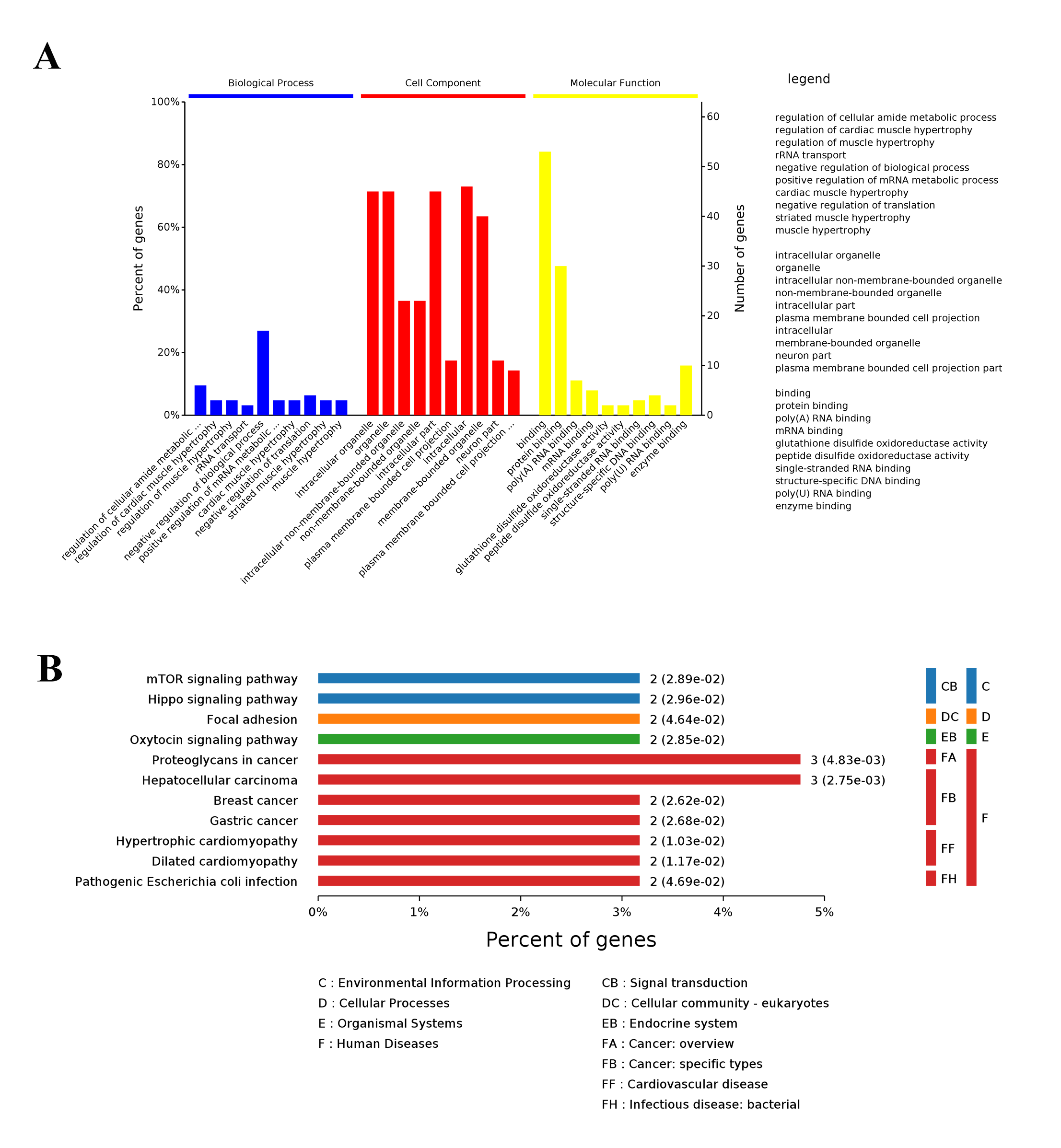

Supplement: Supplementary file 5 — Supplementary Material 5. Supplementary Fig. 3. GO and KEGG pathway analysis of circZBTB46-interacting proteins. [file 12979_2023_386_MOESM5_ESM.tif]

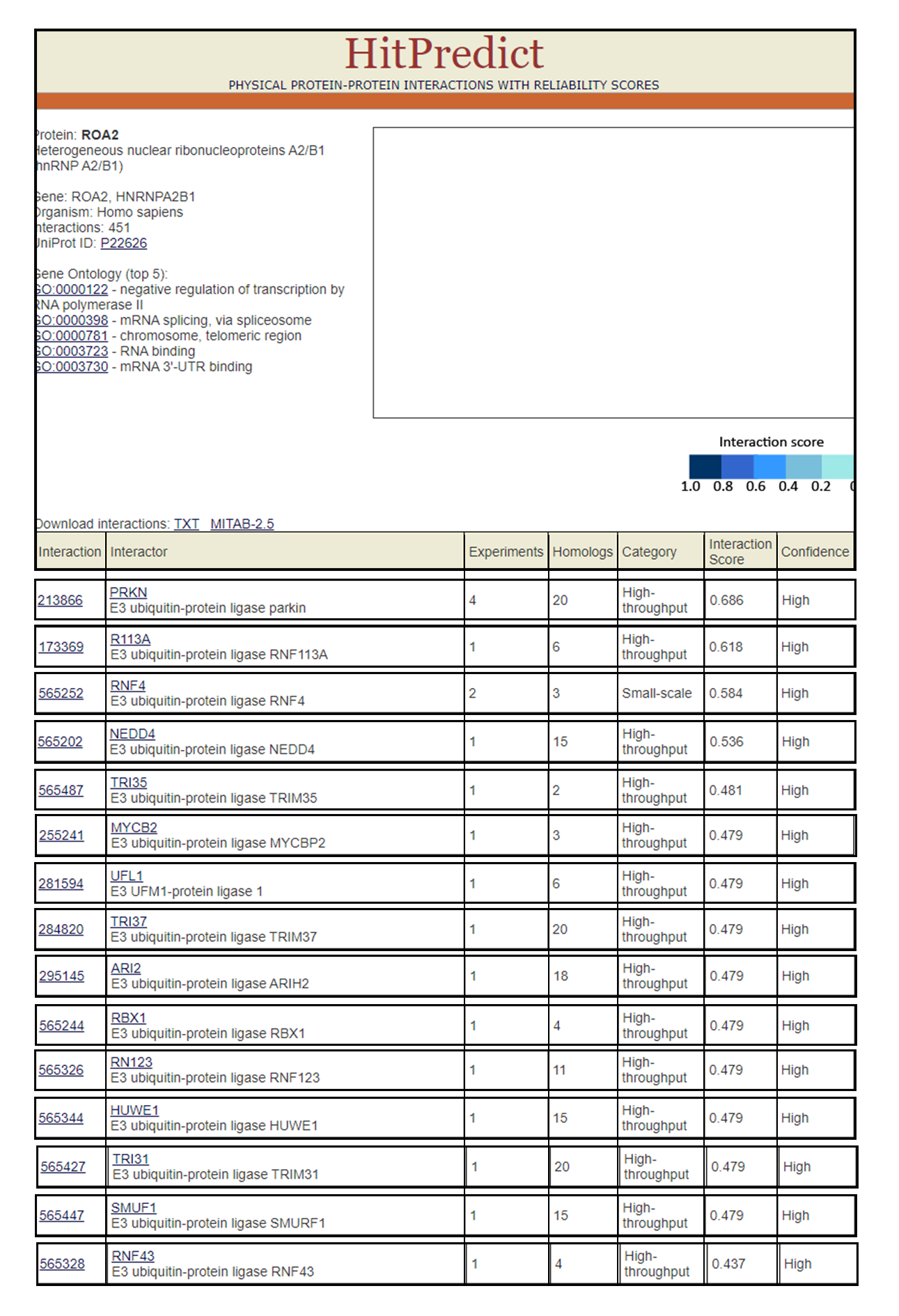

Supplement: Supplementary file 6 — Supplementary Material 6. Supplementary Fig. 4. Potential E-3 ubiquitin-protein ligases of hnRNPA2B1 predicted by the HitPredict Database. [file 12979_2023_386_MOESM6_ESM.tif]
